# Acute respiratory failure revealing a multilocular thymic cyst in an infant: a case report

**DOI:** 10.1186/1757-1626-2-9109

**Published:** 2009-11-30

**Authors:** Bouziri Asma, Khaldi Ammar, Menif Khaled, Guandoura Najoua, Ben Jaballah Nejla

**Affiliations:** 1 Children's Hospital of Tunis, Baab Saadoun 1007 jabbari, Tunis, Tunisia

## Abstract

**Introduction:**

Multilocular thymic cysts are rare benign lesions of the neck and mediastinum that can occur at any age. In children, multilocular thymic cysts are usually symptomatic after the age of 2 years and produce few symptoms. We present an unusual case of a multilocular thymic cyst diagnosed in a 3-month-old girl and causing severe respiratory failure.

**Case presentation:**

A 3 month-old-girl, with a medical history of dyspnea and wheezing since the age of 20 days, presented in our pediatric intensive care unit for acute respiratory failure requiring mechanical ventilation. The chest radiograph showed thoracic distension without any other abnormalities. The diagnosis of severe asthma was initially suspected and the patient was treated by intravenous corticosteroids and continuous perfusion of salbutamol without any improvement. A chest tomography scan was performed and demonstrated an anterior mediastinal multiseptated cystic mass extending from the inferior face of the thyroid gland to the left cardiophrenic angle. Sternotomy and excision biopsy were planned urgently. The cystic mass was excised completely. The histopathological examination confirmed the diagnosis of a multilocular thymic cyst.

**Conclusion:**

The particularities of our observation are the occurrence of a multilocular thymic cyst in a young infant and its presentation by a severe acute respiratory failure mimicking asthma.

## Introduction

Thymic cysts are relatively rare lesions, accounting for approximately 3% of all anterior mediastinal masses [1,2]. They are classified into two groups: congenital and acquired. The former consists of a unilocular cyst with a thin, translucent wall. In contrast, the latter, called a multilocular thymic cyst (MTC) had thick walls and is associated with inflammation. In children, MTCs are less common than congenital thymic cysts [1]. They are usually discovered after the age of 2 years and presented as a cervical mass or remained asymptomatic with a fortuitous discovery on routine chest radiograph [1]. We report an original case of a MTC with an early onset of symptoms at the age of 20 days and an unusual presentation by a severe respiratory failure mimicking asthma due to the compression of the bronchi by the mediastinal mass.

## Case presentation

**Figure 1:**
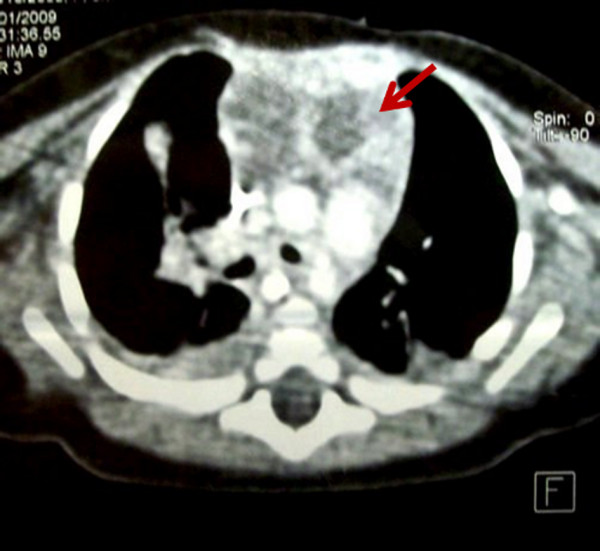
**Chest computed tomography scan showing an anterior mediastinal multiseptated mass with areas of lower density (arrow)**.

**Figure 2:**
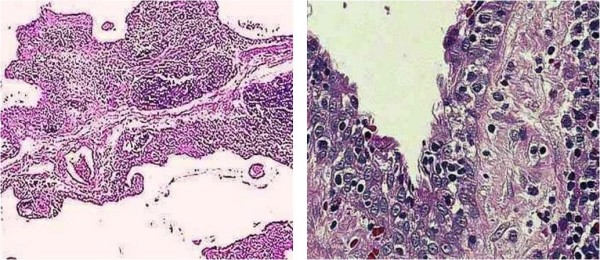
**A: Histopathological examination showing cystic spaces separated by thick walls containing dense lymphoid tissue; B: High-power examination showing the stratified squamous lining of the cyst**.

A Tunisian 3 month-old-girl presented in our pediatric intensive care unit with severe dyspnea causing acute respiratory failure. She suffered from dyspnea and wheezing since the age of 20 days and was treated unsuccessfully by corticosteroids. On physical examination, the patient was agitated and had irregular respiratory rhythm with sibilants in the auscultation. The remainder of the physical examination was normal. The chest radiograph showed thoracic distension without any other abnormalities. The patient was immediately intubated and mechanically ventilated. The diagnosis of severe asthma was suspected and the patient was treated by intravenous corticosteroids and continuous perfusion of salbutamol without any improvement. A right percutaneous jugular central venous catheter was inserted to administer sedation and salbutamol. During the insertion, the puncture of the cervical region returned 20 ml of a yellow exsudative liquid. A cervicomediastinal ultrasonography was performed and showed a large hypoechogenic solid mass of the superior mediastinum. The chest computed tomography scan (CT-scan) (Figure 1) demonstrated an anterior mediastinal multiseptated cystic mass extending from the inferior face of the thyroid gland to the left cardiophrenic angle. β-human chorionic gonadotropin and alpha-fetoprotein markers were within normal limits. Human immunodeficiency virus serology was negative. Sternotomy and excision biopsy were planned urgently because of the critical respiratory state of the child. At surgery, we found a large extrapericardial polylobulated cystic mass contiguous with the thymus and lying laterally on the lungs that she compresses. The cystic mass was excised completely and didn't show any adhesions to the adjacent mediastinal structures. Macroscopically, the specimen measured 7 × 6 × 2.5 cm. On cut section, the cyst showed multiloculated cavities with thick gray walls, and was filled with turbid and blood-stained fluid. The histopathological examination confirmed the diagnosis of MTC and showed several cystic spaces separated by thick walls containing dense lymphoid tissue and foci of cholesterol cleft deposition. Close high-power examination of these foci showed scattered small squamous epithelial pearls consistent with Hassal's corpuscles (Figure 2A and 2B). No postoperative complications occurred. The infant was weaned from ventilation on day 7 after surgery. She was discharged 5 days after extubation and was moderately dyspneic at discharge. Five months later, she was asymptomatic with a normal chest magnetic resonance imaging (MRI).

## Discussion

MTC is a distinctive thymic lesion that morphologically and pathogenetically differs from congenital thymic cyst. The latter is more common and derived from the third pharyngeal pouch. It is unilocular, thin-walled, and lacks inflammatory processes [3]. In contrast, MTC is always multilocular, and accompanied by inflammation. Its wall is thick and fibrous, with accompanying hemorrhage, reactive lymphoid hyperplasia and it contains usually turbid fluid, as in this case. MTC is believed to be the result of hyperplastic and cystic changes of thymic epithelium in response to an underlying inflammatory process of known or unknown cause [1,3]. Thus, MTC has been documented in adult and pediatric patients with mediastinal tumors such as teratoma, lymphoma and thymic carcinoma [4,5] and exceptionally in association with Langerhans' cell histiocytosis of the thymus [2]. Additionally, MTC can be encountered in close association with underlying inflammation of thymic parenchyma and was thus described in patients with various autoimmune disorders such as systemic lupus erythematosus, rheumatoid arthritis, Hashimoto thyroiditis, and Sjögren's syndrome [1,3]. MTCs were also related to human immunodeficiency virus infection [6] and rarely to trauma during surgery [7]. In our observation, HIV serology was negative and there aren't any histopathological signs of malignancy. The inflammatory process causing the MTC-changes in our patient remained unknown. After a review of the English literature available at The Entrez Pubmed database concerning the age of diagnosis and the clinical presentation of thymic cysts in children, we found that congenital thymic cysts were reported previously in infants and newborns and could be symptomatic causing wheezing and upper respiratory infection [8]. However, for MTCs, the youngest age of children reported was 8 months [7] and all cases had few symptoms or were asymptomatic with fortuitous discovery. To our knowledge, this is the first report in the available English literature of a MTC discovered at the age of 3 months and causing severe acute respiratory failure mimicking asthma due to the compression of bronchi by the mediastinal mass. Radiological findings for MTCs are not specific, indicating the importance of pathological investigation for accurate diagnosis [1]. MTCs are composed of multiple cystic cavities or loculi lined by several types of epithelium, including squamous, columnar, cuboidal, and ciliated. The loculi often contain non neoplastic thymic tissue within their walls, associated with chronic inflammation, fibrosis, hemorrhage, cholesterol granuloma formation (Hassal's corpuscles), necrosis, and dystrophic calcification [1]. Treatment for MTCs consists of surgical excision [2]. Postoperative irradiation or chemotherapy is not necessary [2]. MTC may recur postoperatively. Suster and Rosai reported that two of 24 MTCs recurred 2 and 4 years after excision, and that re-excision was successfully performed without evidence of disease 3 and 8 years later. It is thought that the 2 recurrent cases may have been due to incomplete resection of MTCs [1]. After 5 months of follow up, our patient was asymptomatic with a normal MRI.

## Conclusion

This report illustrates an unusual case of a MTC revealing by a severe acute respiratory failure requiring mechanical ventilation in a 3-month-old infant. Our case is original in terms of clinical presentation and age of diagnosis.

## Consent

Written informed consent was obtained from the parents of the patient for publication of this case report and accompanying images. A copy of the written consent is available for review by the Editor-in-Chief of this journal.

## Competing interests

The authors declare that they have no competing interests.

## Authors' contributions

BA, MK, GN and BN were major contributors in writing the manuscript.

KA collected the histological and radiological images.
